# Impairment of synaptic plasticity in the primary somatosensory cortex in a model of diabetic mice

**DOI:** 10.3389/fncel.2024.1444395

**Published:** 2024-07-30

**Authors:** Nuria García-Magro, Alberto Mesa-Lombardo, Natali Barros-Zulaica, Ángel Nuñez

**Affiliations:** ^1^Department of Anatomy, Faculty of Health Science, Universidad Francisco de Vitoria, Pozuelo de Alarcón, Madrid, Spain; ^2^Department of Anatomy, Histology and Neuroscience, Medical School, Autónoma University of Madrid, Madrid, Spain; ^3^Blue Brain Project, Ecole Polytechnique Fédérale de Lausanne, Campus Biotech, Geneva, Switzerland

**Keywords:** synaptic plasticity, IGF-I, GluR1 receptor, NMDA receptor, PI3-kinase, mTOR, somatosensory cortex, diabetes

## Abstract

Type 1 and type 2 diabetic patients experience alterations in the Central Nervous System, leading to cognitive deficits. Cognitive deficits have been also observed in animal models of diabetes such as impaired sensory perception, as well as deficits in working and spatial memory functions. It has been suggested that a reduction of insulin-like growth factor-I (IGF-I) and/or insulin levels may induce these neurological disorders. We have studied synaptic plasticity in the primary somatosensory cortex of young streptozotocin (STZ)-diabetic mice. We focused on the influence of reduced IGF-I brain levels on cortical synaptic plasticity. Unit recordings were conducted in layer 2/3 neurons of the primary somatosensory (S1) cortex in both control and STZ-diabetic mice under isoflurane anesthesia. Synaptic plasticity was induced by repetitive whisker stimulation. Results showed that repetitive stimulation of whiskers (8 Hz induction train) elicited a long-term potentiation (LTP) in layer 2/3 neurons of the S1 cortex of control mice. In contrast, the same induction train elicited a long-term depression (LTD) in STZ-diabetic mice that was dependent on NMDA and metabotropic glutamatergic receptors. The reduction of IGF-I brain levels in diabetes could be responsible of synaptic plasticity impairment, as evidenced by improved response facilitation in STZ-diabetic mice following the application of IGF-I. This hypothesis was further supported by immunochemical techniques, which revealed a reduction in IGF-I receptors in the layer 2/3 of the S1 cortex in STZ-diabetic animals. The observed synaptic plasticity impairments in STZ-diabetic animals were accompanied by decreased performance in a whisker discrimination task, along with reductions in IGF-I, GluR1, and NMDA receptors observed in immunochemical studies. In conclusion, impaired synaptic plasticity in the S1 cortex may stem from reduced IGF-I signaling, leading to decreased intracellular signal pathways and thus, glutamatergic receptor numbers in the cellular membrane.

## 1 Introduction

Diabetes mellitus (DM) type 1 is characterized by high a blood glucose level due to an immune-mediated destruction of pancreatic β-cells. Type 1 and type 2 diabetic patients exhibit metabolic and vascular disorders, which additionally lead to alterations in the Central Nervous System (CNS), resulting in cognitive deficits (Ryan, 1988; Strachan et al., 1997; Gispen and Biessels, 2000). This decline is increasingly recognized as an important comorbidity of DM (Biessels and Despa, 2018). Diabetic patients frequently show an increase of the latency of sensory evoked potentials, suggesting alterations in the CNS (Di Mario et al., 1995). While cognitive deficits may be modest in young adults, they can be severe in the older populations (Stewart and Liolitsa, 1999; Saedi et al., 2016; Li et al., 2017). Reduced levels of insulin-like growth factor-I (IGF-I) and/or insulin have been proposed as potential causes of these neurological disorders because IGF-I application reduces cognitive deficits (Ishii, 1995; Guo et al., 1999; Lupien et al., 2003). Insulin treatment in animal models of diabetes prevents learning deficits when given at the onset of diabetes. However, when insulin is given later (10 weeks after diabetes induction), only a partial improvement was observed (Biessels et al., 1999).

Streptozotocin (STZ)-induced diabetic mice and rats, also develop cognitive deficits (Flood et al., 1990; Biessels et al., 1996; Kamal et al., 2000). STZ is widely used as a type 1 diabetes mellitus (T1DM)-inducer in experimental animals, through its toxic effect on insulin producing β-cells. In addition to a reduction in insulin levels, brain IGF-I levels are also reduced (Ishii, 1995; Busiguina et al., 1996). T1DM mice induced by STZ injection (STZ-diabetic mice) show impaired sensory perception and working and spatial memory functions (Saedi et al., 2016; Rostami et al., 2017; Momeni et al., 2021). Somatosensory responses are altered during the first month of diabetes (Courteix et al., 1993; Artola, 2013).

Previous works have reported changes in synaptic plasticity that could explain the cognitive deficits that occur in DM (Strachan et al., 1997; Gispen and Biessels, 2000; Valastro et al., 2002; Artola et al., 2005). We reported a reduction of the sciatic nerve evoked potential amplitude in the primary somatosensory (S1) cortex at 8 weeks of diabetes that was accompanied by a decrease in GluR2/3 AMPA receptor subunit, suggesting that synaptic plasticity was altered in the cortex (Piriz et al., 2009). It has been reported that long-term potentiation (LTP) is also decreased in the hippocampus of STZ-diabetic mice and rats probably due to a reduction of the expression of NMDA receptors (Chabot et al., 1997; Kamal et al., 1999; Artola et al., 2005; Artola, 2013), concretely a downregulation of NR1 and NR2A subunits (Sasaki-Hamada et al., 2012; Wang et al., 2022). The affinity of glutamate for AMPA is also reduced in STZ-diabetic rats (Gagne et al., 1997). However, other authors have found an up-regulation of glutamatergic receptors in the hippocampus of diabetic mice although LTP in the hippocampus was reduced respect to control animals (Valastro et al., 2002). In contrast to LTP, expression of long-term depression (LTD) is enhanced in the CA1 after low-frequency stimulation of hippocampal slices from diabetic rats (Kamal et al., 1999), suggesting that synaptic plasticity deficits in T1DM may involve changes in the membrane excitability or in the synaptic receptors involved in the induction of LTP and LTD.

Therefore, DM can exacerbate the cognitive deterioration that mainly occurs in the elderly. The following experiments were conducted to characterize the influence of DM on somatosensory processing in the S1 cortex of young streptozotocin (STZ)-induced diabetic mice. We have focused on the impact of reduced brain levels of IGF-I, which occurs in DM, on synaptic plasticity. Unit recordings in layer 2/3 neurons of the S1 cortex were performed in control and in STZ-diabetic mice that were anesthetized with isoflurane. Synaptic plasticity was induced by repetitive stimulation of the whiskers, since we have previously published that a repetitive induces a long-lasting response potentiation (Barros-Zulaica et al., 2014; Garcia-Magro et al., 2022). We have additionally used immunological and behavioral techniques to study the change in synaptic plasticity observed in STZ-diabetic mice.

## 2 Materials and methods

### 2.1 Animals

Experiments were carried out on young adult male C57BL/6J mice were used (*n* = 58; 3 month old; weight: 22-28g. Harlan Laboratories, Spain). Mutant mice with low serum IGF-I (LID mice; 3 months old; 22–28 g) were obtained from the Cajal Institute. To generate these mutant mice, the Cre/loxP recombination system was used to eliminate the IGF-I gene exclusively in the liver. Deletion of the IGF-1 gene in the liver abolished IGF-I mRNA expression and caused a dramatic reduction in circulating IGF-I levels (Yakar et al., 1999). Mice were separated in: STZ-diabetic mice (*n* = 33) and control mice (*n* = 25) that only received the vehicle. All mice were housed under a 12:12-h dark/light cycle at 22 ± 2°C, with *ad libitum* access to food and water. All animal procedures were carried out in accordance with the European guidelines (2010/63, European Council Directives) and the Ethical Committee of the Autonomous University of Madrid and Government of the Community of Madrid (PROEX: 181.6/21). Efforts were made to minimize animal suffering and to reduce the number of mice used.

### 2.2 STZ-dependent diabetes

To induce diabetes in our mouse population, we used the protocol published elsewhere (Perez-Taboada et al., 2020). Briefly, STZ (50 mg/kg, i.p., Merck, Spain) was injected for 5 consecutive days. After 4 h of fasting, glucose levels were controlled using tail blood and through a glucometer (Glucoleader-Yasee GLM-76, Nessler, Spain). Glucose levels were measured before the STZ injection and every 7 days during the three-week development of diabetes. When glucose levels were > 300 mg/dL they were considered diabetic. Control mice were administered with the vehicle (10 mM sodium citrate, 0.9% NaCl, pH 4.5, i.p.). A double-blinding strategy was implemented throughout all stages of the experiment. A technician was responsible for measuring glucose levels and caring for the animals, while the researcher conducted behavioral and anatomical studies, or electrophysiological recordings without knowing the group to which the mouse belonged.

### 2.3 Recordings and tactile stimulation

Isoflurane anesthetized mice (2% induction, 1–1.5% maintenance doses) were placed in a David Kopf stereotaxic frame (Tujunga, CA, United States) where body temperature was stablished at 37°C (Gaymar T/Pump water-heated pad; Orchard Park, NY, United States). The skull was exposed, and a craniotomy was opened over the barrel S1 cortex [coordinates from Bregma: 0.5–2 mm AP; 3–4 mm L; Paxinos and Franklin (2003)] to introduce a tungsten microelectrode (2–5 MΩ, A-M Systems, United States) for unit recording at the level of 2/3 layer (deep 200–500 μm). Recordings were performed in both hemispheres. Recordings were filtered (0.3–3 kHz), amplified (DAM50; World Precision Instruments, Sarasota, United States) and fed into a personal computer (sample rate: 10 kHz) for off-line analysis (Spike2 software; Cambridge Electronic Design (CED), Cambridge, United Kingdom).

Tactile stimulation was delivered by an pneumatic pressure pump that generate brief air-pulses of 20 ms duration direct to the whiskers (Picospritzer, Hollis, NH, USA; 1–2 kg/cm2). Experimental procedures consisted of air pulses at 0.5 Hz lasting 10 min for basal recording. A stimulation train of air pulses at 8 Hz for 10 s was used to induce synaptic plasticity. This stimulation frequency is within the range of frequencies that rodents use to explore the environment [4–12 Hz; see for review (Moore, 2004)]. After that, the whisker was stimulated for 30 min at 0.5 Hz.

### 2.4 Drugs

A 1 μL Hamilton syringe was used to deliver drugs on the S1 cortex. IGF-I (5 or 10 nM, 0.2 μL; Pre-Protech, United States), the NMDA-receptor antagonist AP5 (50 μM; 0.2 μL; Merck, Spain), the non-selective antagonist of metabotropic glutamate receptors (MCPG; 1 mM; 0.2 μL), the non-selective phosphoinositol kinase inhibitor Wortmannin (50 μM, 0.2 μL, Merck, Spain) or the selective mTOR complex inhibitor Rapamycin (2 mM, 0.2 μL, Merck, Spain) were applied.

### 2.5 Immunohistochemistry

Animals were deeply anesthetized with an overdose of sodium-pentobarbital (50 mg/kg i.p.) and then perfused transcardially with saline followed by formaldehyde solution (4% in 0.1 M phosphate buffer (PB); pH 7.4). Coronal brain sections were cut in a sliding microtome (40-μm-thick; Leica SM2400, Leica Biosystems, Nussloch) and placed in PB 0.1 M. Sections were incubated in PBS 0.02 M, 1%Triton X-100, NDS 10% (blocking solution), followed by 24 h incubation at 4°C with primary antibodies (1:500) in blocking solution. In a series of sections, the mouse monoclonal IGF-I Receptor antibody (Santa Cruz, CA, United States, sc-27160) and rabbit anti-glutamate Receptor 1 (Abcam, Cambridge, United Kingdom, ab31232) were used. In another series of sections, the mouse anti N-methyl-D-aspartate receptor subunit NR1 (anti-NMDA Receptor 1; Abcam, Cambridge, United Kingdom, ab193310) was also used. After washing in PBS, Alexa-coupled donkey anti rabbit and donkey anti-mouse polyclonal secondary antibodies (1:200, Molecular Probes, Eugene, United States) were used. Finally, slices were incubated in a 1:3000 dilution in PB 0.1M of Hoechst for 5 min. After that, they were rinsed several times in PB 0.1M, mounted with gerbatol mounting medium. The omission of the primary antibody was used as a control.

Confocal images were obtained and processed as we published elsewhere (Garcia-Magro et al., 2022). Briefly, a TCS SP5 Spectral Leica confocal microscope (Leica; Wetzlar, Germany) was used to obtain images of the S1 cortex, using a 20X or 63X oil immersion objectives. Leica LAS AF software was utilized to get image stacks of 1024 × 1024, which were collapsed to generate TIFF files with the projections of maximum intensity using a thickness of 10 microns of tissue in all cases. Images were converted to 8-bit grayscale using ImageJ image analysis software for Windows (Microsoft; Albuquerque, NM, United States). The same image processing was applied in all cases, limiting itself to minor adjustments to grayscale and brightness to improve viewing. A densitometric analysis of IGF-1R, GluR1 and NMDAR1 immunoreactivity was performed through ImageJ’s software. We used five sections to obtain the mean value of optical density for each animal, with four animals included in each immunoreactivity study. Values were analyzed with Graph Pad Prism 10 software (San Diego, CA, United States).

### 2.6 Behavioral experiments

To assess the mice’s ability to distinguish different textures using their whiskers, we employed the two-trial Y-maze test (Dellu et al., 2000; Noriega-Prieto et al., 2021). The Y-maze consisted of three arms, each measuring 25 cm in length, 5 cm in width, and 14 cm in height, two of which were covered with 500-grit sandpaper (familiar texture), while the third arm was covered with 220-grit sandpaper (novel texture) Since the maze arms were identical and lacked external cues, the mouse’s discrimination between novelty and familiarity relied solely on the different textures that could be sensed through its whiskers. Initially during acquisition phase, the mouse was randomly placed in one of the familiar arms to explore arms for 5 min; the third arm (novel) was closed. After the first trial, the mouse was returned to the home cage for 5 min. In the retrieval phase, the mouse was placed back in the same arm where it started during the acquisition phase and given 5 min to freely explore all three arms. To eliminate any potential olfactory cues, 70% ethanol was used to clean the maze between trials. To measure the time spent exploring the novel arm, an offline video analysis was performed.

### 2.7 Statistical analysis

**Figure 1:**
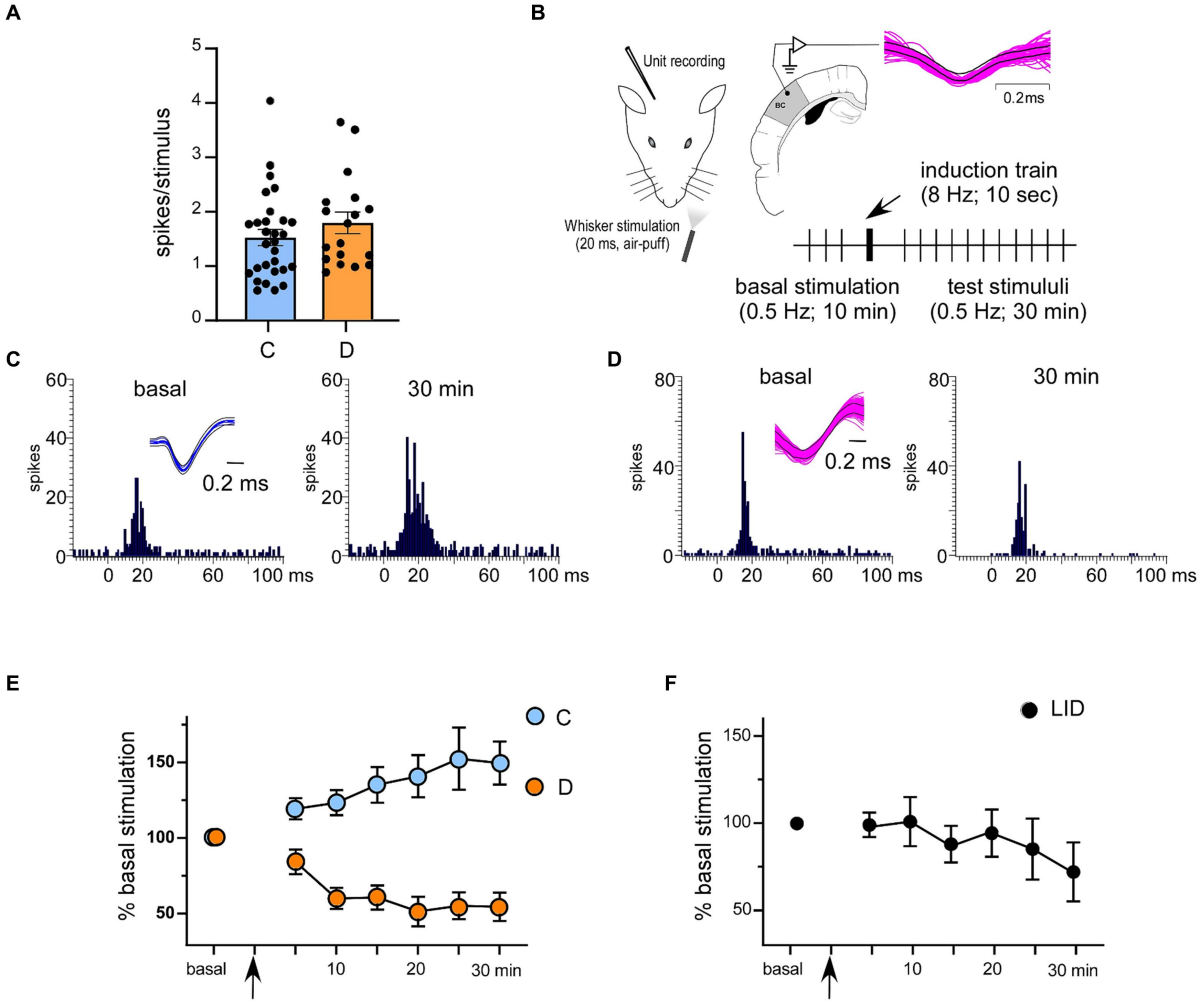
Response characteristics of 2/3 cortical neurons in control and in STZ-diabetic mice. **(A)** Basal responses (spikes/stimulus) in control and STZ-diabetic mice. No differences were observed. **(B)** The stimulation protocol to induce synaptic plasticity is shown. Superimposed waveforms of spikes are shown in the inset; the equal waveform indicates that recorded spikes are from the same cell. **(C,D)** Representative PSTHs in basal condition (left histogram) and 30 min after the stimulation train (right histogram) in a control and in a STZ-diabetic mice, respectively. Insets show superimposed waveforms of spikes. **(E)** Application of the induction train (8 Hz for 10 s) induced LTP in control mice or LTD in STZ-diabetic mice. **(F)** The same induction train in the serum IGF-I deficient mice (LID mice) elicited LTD. In this and in the following figures **C** means control animals and **D** means STZ-diabetic animals.

Single-unit activity was extracted with Spike2 software for spike waveform identification and analysis. Recordings were accepted for statistical analysis when the spike amplitude fluctuations were lower than 10% throughout the experiment. Single units were discriminated by threshold spike detection and by waveform analysis tool of Spike 2 software (see Figure 1B). In some cases two neurons could be extracted from the same recording because their amplitude and waveform were clearly differentiated. The selected spikes were stored at a 1-ms resolution, and isolated single-units were analyzed and quantified.

The peristimulus time histograms (PSTHs) were calculated to quantify whisker responses (1 ms bin-width; 50 ms post-stimulus time window). The average whisker response during the basal condition was set to a baseline of 100%. The effect of the induction train (8 Hz; 10 s) was calculated for 30 min. To perform statistical analysis, we used Graph Pad Prism 10 software (San Diego, CA, United States). Any differences between the variables were compared using two-way parametric tests (Student’s t-test or paired t-test) following normality testing with the Kolmogorov–Smirnov test. We have also used the repeated measures (RM) version of the two-way ANOVA. Data were presented as the mea*n* ± standard error of the mean (SEM), with n indicating the number of mice per group for a given experiment or the number of neurons analyzed. The results were considered significant at *p* < 0.05 (*p < 0.05, **p < 0.01, ***p < 0.001).

## 3 Results

### 3.1 STZ-diabetic mice showed impaired of the synaptic plasticity

Neurons were recorded from layer 2/3 of the S1 cortex in 12 controls or 13 STZ-diabetic mice. Neurons from both animal groups were silent or had a low firing rate (0.35 ± 0.11 Hz and 0.29 ± 0.18 Hz; *n* = 28 neurons and 17 neurons in control or STZ-diabetic mice, respectively). All selected neurons displayed a response to contralateral displacements of one or two whiskers. The mean whisker response was 1.51 ± 0.14 spikes/stimulus (*n* = 28 neurons) and 1.78 ± 0.2 spikes/stimulus for control or STZ-diabetic mice (*n* = 17 neurons; Figure 1A; Table 1). No significant differences (*p* > 0.05) were observed in the spontaneous activity or in the number of spikes evoked by the stimulus between both animal groups. The recorded neurons included in this study may be pyramidal cells because they exhibited a low firing rate, a small receptive field (one or two whiskers), and a reduced whisker response, as previously reported (de Kock et al., 2007; de Kock and Sakmann, 2008; Chakrabarti and Alloway, 2009; Petersen, 2019).

**Table 1:** Response characteristics of S1 cortical neurons in basal and 30 min after the induction train.

	Spikes/stimulus (mea*n* ± SEM)		Number of neurons		
	Basal	After induction train	LTP	LTD	–
Control mice	1.51 ± 0.14	2.25 ± 0.32*	23	1	4
STZ-diabetic mice	1.78 ± 0.2	0.95 ± 0.13***	2	11	4
LID mice	1.51 ± 0.18	1.05 ± 0.24	1	7	2
C + Wortmannin	1.21 ± 0.12	0.92 ± 0.36	1	3	1
C + Rapamicine	1.45 ± 0.16	0.99 ± 0.2**	0	8	2
D + AP5	1.24 ± 0.15	1.34 ± 0.19	5	3	2
D + MCPG	1.38 ± 0.14	1.13 ± 0.17	4	6	3

C, control animals; D, STZ-diabetic animals. *p< 0.05, **p< 0.01, ***p< 0.001.

Previous studies have shown that repetitive stimulation of the whiskers induces a long-lasting response potentiation (LTP) that lasts for at least 30 min (Barros-Zulaica et al., 2014; Garcia-Magro et al., 2022). In the present work, we tested whether LTP evoked by a repetitive stimulation was altered in STZ-diabetic mice. After 10 min of basal whisker stimulation (0.5 Hz; the mean value was considered 100%; basal response), we applied a stimulation train at 8 Hz for 10 s (induction train) to mimic the exploratory behavior of mice. After that, we maintained the 0.5 Hz stimulation for 30 min (Figure 1B). In control animals, layer 2/3 neurons increased up to 149.9 ± 12.2% their mean response at 30 min after the stimulation train (1.51 ± 0.14 to 2.25 ± 0.32 spikes/stimulus; *n* = 28 neurons; *p* = 0.013, respect to basal response; Table 1). We termed this increase in the whisker response as long-term potentiation (whisker-evoked LTP; Figure 1E). The PSTHs showed an increase in the number of evoked spikes and in the response duration (Figure 1C). To determine the proportion of neurons that were facilitated or depressed by the induction train, we establish a threshold of 10%. Thus, neurons exhibiting LTP were defined as those whose responses were > 110% 30 min after the induction train; neurons displaying LTD were defined as those whose responses were < 90% 30 min after the induction train, while those unaffected were also noted. With this criterion, in control mice 23 out of 28 neurons showed LTP (82.5%), one neuron showed LTD (3.5%) and four neurons were not affected by the induction train (14%; Table 1).

In contrast to control animals, layer 2/3 neurons from STZ-diabetic animals decreased their response to 53.3 ± 6.8% (1.78 ± 0.2 to 0.95 ± 0.13 spikes/stimulus; *n* = **17** neurons; *p* = 0.0005, respect to basal value) 30 min after the induction train (Figure 1E; Table 1). We termed this decrease in the whisker response as long-term depression (whisker-evoked LTD). PSTHs showed a reduction of the number of spikes evoked by the whisker stimulation (Figure 1D). With the same criteria as mentioned above, in diabetic mice two out of **17** neurons showed LTP (6%), 11 neurons showed LTD (69%) and four neurons were not affected (25%; Table 1). The time course in control mice was statistically significant when compared with that in diabetic mice [two-way RM ANOVA *F* (1, 329) = 67.40 *p* < 0.001***].

It is known that diabetic patients and animal models of diabetes show reduced IGF-I levels that have been postulated as a possible cause of neurological disorders (Ishii, 1995; Busiguina et al., 1996; Guo et al., 1999; Ishii and Lupien, 2003). For this reason, we tested the ability to induce response facilitation in a mouse model of serum IGF-I deficiency (the LID mouse; 6 animals) that show a reduced brain IGF-I input (Trueba-Saiz et al., 2013). Repetitive stimulation of whiskers in LID mice induced a reduction of the response (LTD), as also occurred in STZ-diabetic mice. Neurons recorded from LID animals decreased their response to 69.5 ± 7.9% (1.51 ± 0.18 to 1.05 ± 0.24 spikes/stimulus; *n* = 10 neurons; *p* = 0.1341; respect to basal value) 30 min after the induction train (Figure 1F; Table 1). The time course in control mice was statistically significant compared with LID mice [two-way RM ANOVA *F* (1, 231) = 10.31 *p* = 0.0015 **]. In this case, one neuron showed LTP (10%), seven neurons showed LTD (70%) and two neurons were not affected (20%).

### 3.2 Mechanisms of the whisker-evoked LTD

**Figure 2:**
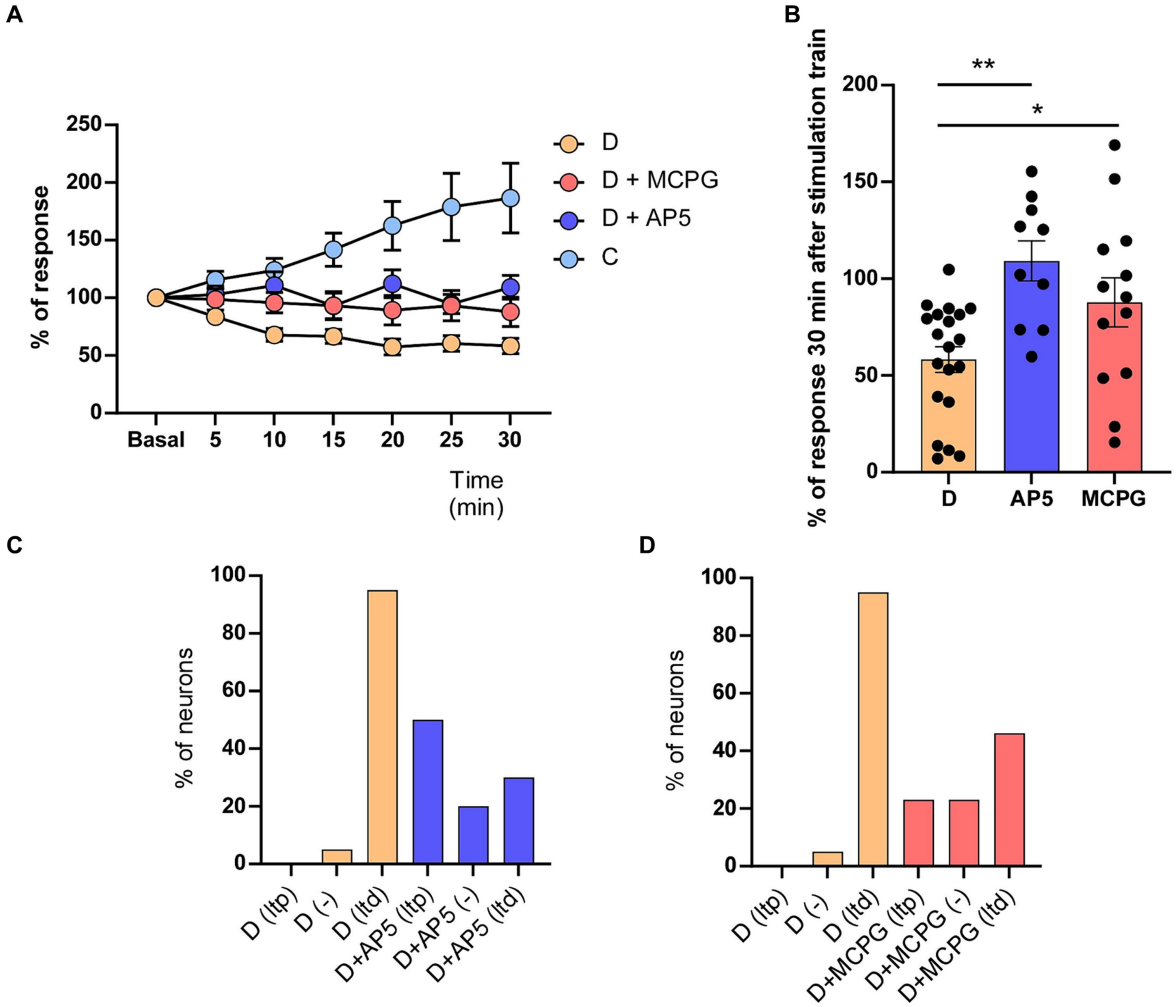
AP5 or MCPG affects whisker-evoked LTD in STZ-diabetic mice. **(A)** Unlike neurons recorded in control mice that expressed LTP (186.5 ± 30.21%, *n* = 10 neurons), both NMDA receptor antagonist (AP5) or the metabotropic receptor antagonist (MCPG) abolished LTD in STZ-diabetic mice. The time course in STZ-diabetic mice was statistically significant compared with STZ-diabetic mice in presence of AP5 (Two-way RM ANOVA *F* (1, 196) = 66.74 *p* < 0.001) or MCPG (Two-way RM ANOVA *F* (1, 217) = 30.87 *p* < 0.001). **(B)** Comparison of mean % response of STZ-diabetic animals is shown 30 min after the induction train under control conditions and in the presence of AP5 (*p* = 0.0002 ***) or MPCG (*p* = 0.031 *). **(C)** Plot shows the percentage of neurons showing LTP or LTD in STZ-diabetic animals in control conditions or after the application of AP5. **(D)** Plot as in C after the application of MCPG. The application of AP5 or MCPG on the S1 cortex increased the percentage of neurons that show LTP in STZ-diabetic mice.

Most cortical synapses that undergo LTP or LTD use glutamate as their neurotransmitter acting on different receptors. LTP and major forms of LTD require NMDA and group I mGlu receptors (Cho et al., 2000; Collingridge et al., 2010; Rodriguez-Moreno et al., 2013). First, we tested whether the NMDA receptor antagonist AP5 affects whisker-evoked LTD in STZ-diabetic mice. We applied an induction train in the whiskers in control conditions (saline solution was applied on the cortex) and after the application of 0.5 μL of AP5 (50 μM; 6 animals). We measured the response every 5 min for 30 min after the induction train. Control recordings from neurons of diabetic animals (*n* = 20 neurons) showed a progressive decrease in the response (up to 53.3 ± 6.8% of basal values at 30 min after the induction train; LTD; Figures 2A,B), as was indicated above. However, after application of AP5, the response did not decrease, reaching 109.2 ± 10.38% of basal values at 30 min after the induction train (*n* = 10 neurons). In presence of AP5, five of 10 neurons tested showed LTP 30 min after the induction train (50%), three neurons reduced their response (30%; LTD) and two neurons were not affected (20%; Figure 2C; Table 1).

In addition, we also tested the effect of the (RS)-MCPG, a non-selective antagonist of group I/II metabotropic glutamate receptors (mGluR) on the whisker-evoked LTD observed in STZ-diabetic mice. Whisker responses were greatly reduced in neurons recorded after the cortical application of MCPG (1 mM; 0.2 μL; 6 animals), reaching 87.8 ± 12.68% of basal values (*n* = 13 neurons). In presence of MCPG, four of 13 neurons tested showed LTP after the induction train (30.7%), six neurons reduced their response (46.1%; LTD) and were three neurons not affected (23.2%; Figure 2D).

### 3.3 Effect of IGF-I on the whisker-evoked responses

**Figure 3:**
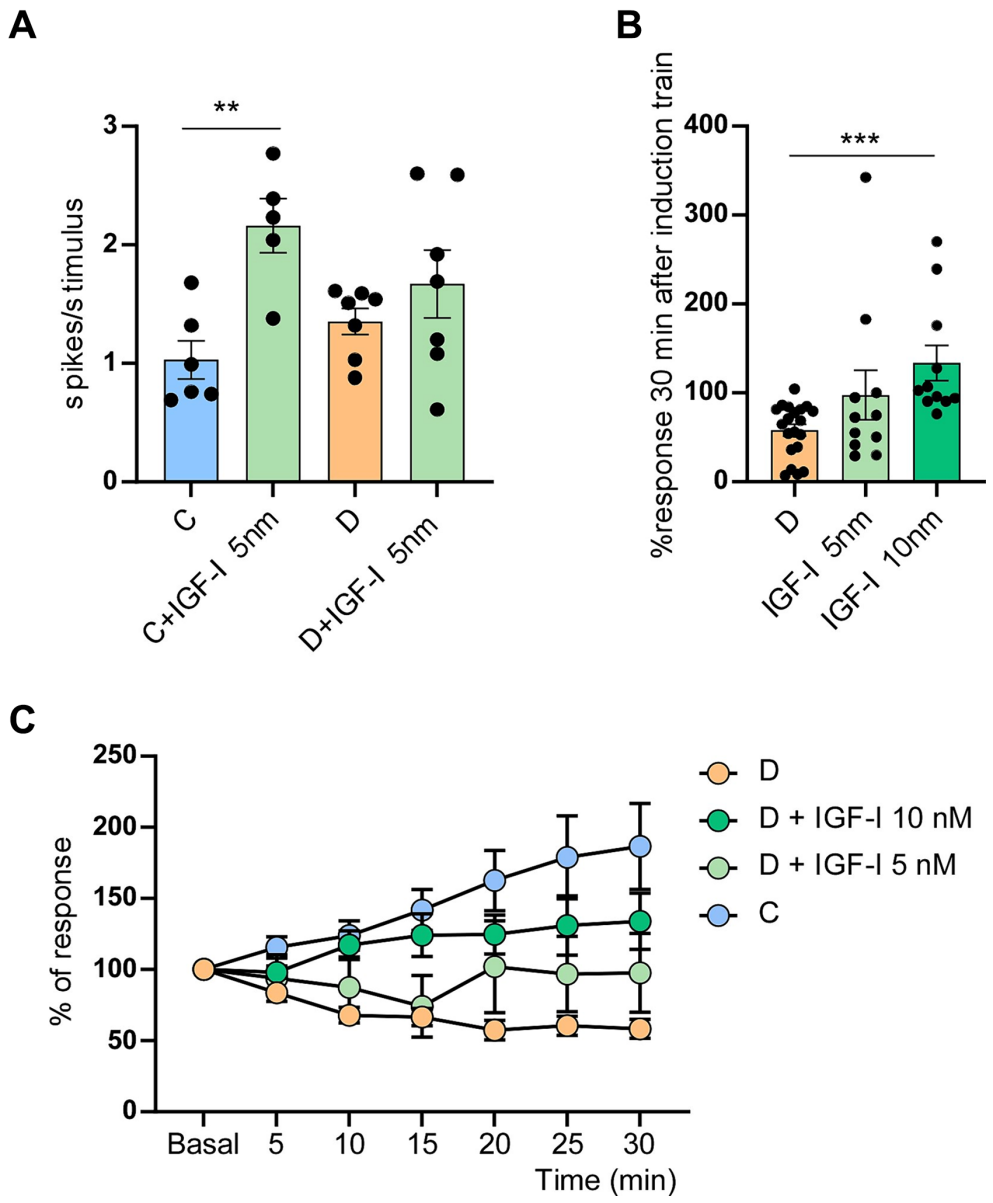
Application of IGF-I blocked the LTD in STZ-diabetic mice. **(A)** Plot shows the mean number of spikes/stimulus in STZ-diabetic animals (orange) or control animals (blue) when IGF-I 5 nM (green) was applied on the cortex. Whisker responses increased by IGF-I only in control animals (*p* = 0.005 **). **(B)** Comparison of % whisker responses respect to basal values in STZ-diabetic mice, measured 30 min after the induction train under control conditions and in presence of IGF-I 5 nM (ns) or IGF-I 10 nM (*p* = 0.0001 ***). IGF-I facilitated whisker responses. **(C)** Time course of responses in control and in STZ-diabetic animals respect to basal values; control values are the same that in Figure 2A (*n* = 10 neurons). Different IGF-I concentrations were applied on the S1 cortex of STZ-diabetic animals. The increase response was statistically significant between neurons of STZ-diabetic animals recorded in control conditions and those recorded in the presence of IGF-I 5 nM [two-way ANOVA *F* (1, 203) = 10.30 *p* = 0.0015] or 10 nM [two-way ANOVA F (1, 203) = 96.77 *p* < 0.001]. Comparison between doses of IGF-I also showed significant differences [two-way ANOVA *F* (1, 140) = 6.16 *p* = 0.014].

IGF-I has been demonstrated to enhance neuronal activity. In the S1 cortex, IGF-I induces an increase in whisker responses by the activation of NMDA receptors (Barros-Zulaica et al., 2014, 2019; Garcia-Magro et al., 2022). In these experiments, IGF-I (5 nM; 0.2 μL) also increased whisker responses in either control (1.0 ± 0.16 to 2.2 ± 0.23 spikes/stimulus; *p* = 0.005; *n* = 6 neurons; 5 animals) or in STZ-diabetic mice (1.3 ± 0. 15 to 1.63 ± 0.72 spikes/stimulus; *p* = 0.33 *n* = 7 neurons; 5 animals; Figure 3A). In presence of low doses of IGF-I (5 nM; 0.2 μL; *n* = 11 neurons; Figure 3B), the 8 Hz-induction train evoked an increase in the response, reaching 97.6 ± 27.76% at 30 min in comparison with baseline values. Considering that in diabetes IGF-I levels are reduced, we have studied whether the local application of IGF-I on the S1 cortex could restore the LTP in STZ-diabetic after the induction train (Figure 3B). To know whether the dose of IGF was important to restore LTP in STZ-diabetic animals, we also used a higher dose of IGF-I (10 nM; 0.2 μL). In this case, the response increased to 133.8 ± 19.8%, partially restoring the LTP (*n* = 12 neurons; 5 animals; Figure 3B).

The difference between the time course of the increasing response was statistically significant in neurons of STZ-diabetic animals recorded in control conditions and those recorded in the presence of IGF-I 5nM [two-way ANOVA *F* (1, 203) = 10.30 *p* = 0.0015 **] and 10 nM [two-way ANOVA F (1, 203) = 96.77 *p* < 0.001***]. Comparison between doses of IGF-I also showed significant differences [two-way RM ANOVA *F* (1, 140) = 6.16 *p* = 0.014 *]. However, results obtained after local IGF-I application did not get the values reached in control animals (compare light blue and green spots with strong blue spots in Figure 3A).

### 3.4 Wortmannin or rapamicine induced LTD in control mice

**Figure 4:**
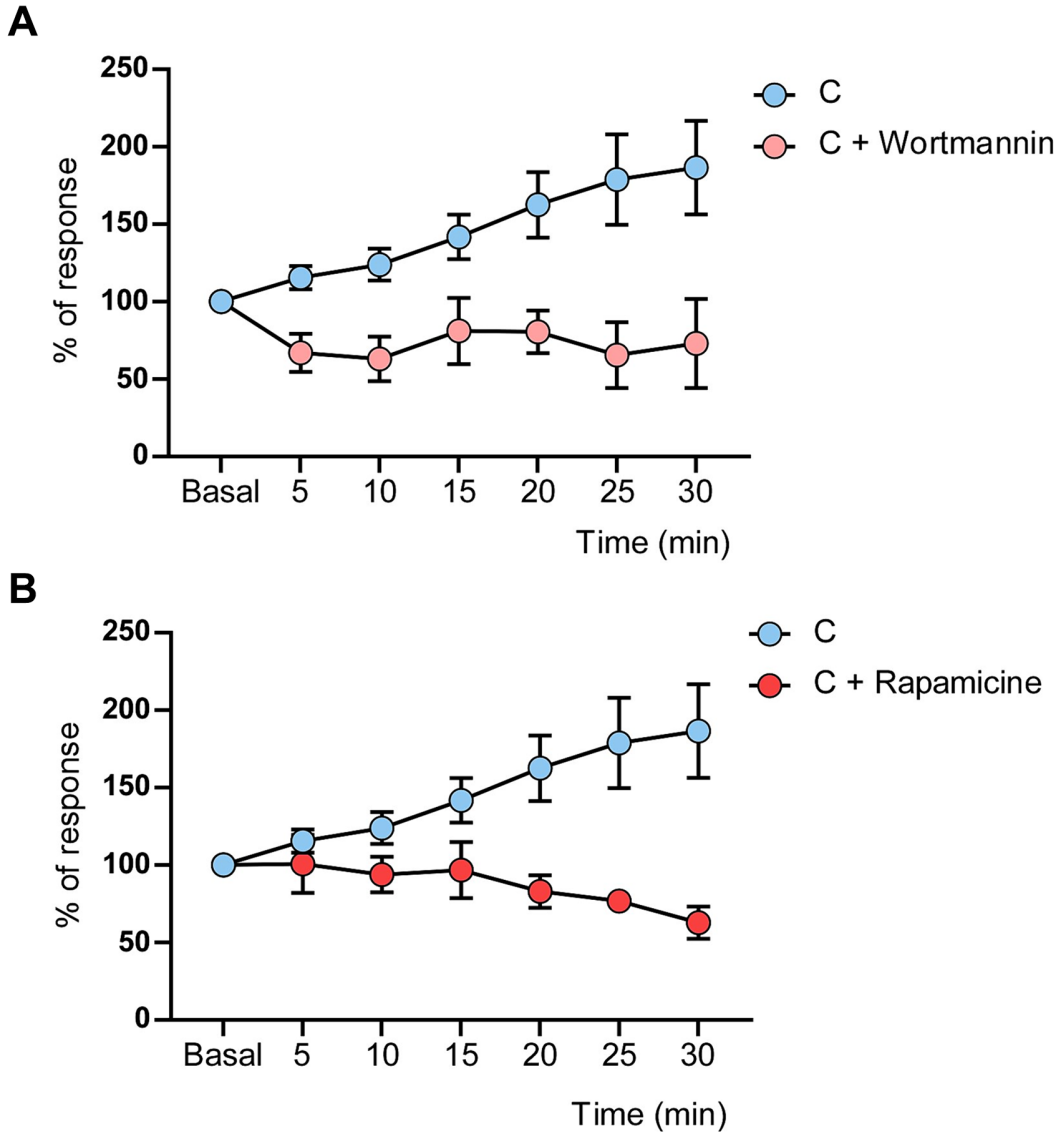
Wortmannin or rapamicine induced LTD in control mice. **(A)** LTD was evoked by the induction train in control mice when wortmannin (50 mM, 0.2 μL) was applied on the cortex. **(B)** LTD was evoked by the induction train in control mice when rapamicine (2 mM, 0.2 μL) was applied on the cortex. Significant differences were found in the response decrease in the presence of Wortmaninn [Two-Way RM ANOVA, group factor: *F* (1, 224) = 14.50 *p* = 0.0002] or rapamycin [Two-Way RM ANOVA, group factor: *F* (1, 259) = 19.56 *p* < 0.0001]. Control values are the same that in Figure 2A (*n* = 10 neurons).

It is known that IGF-I uses the PI3K-AKT–mTOR intracellular pathway to induce different cellular effects (Fernandez and Torres-Aleman, 2012; Nuñez et al., 2023). After blocking the PI3K-AKT–mTOR signaling pathway with local application of wortmannin (a non-selective phosphoinositol kinase inhibitor; 50 mM; 0.2 μL on the S1 cortex; 4 animals; *n* = 5 neurons) or rapamycin (mTOR inhibitor; 2 mM, 0.2 μL on the S1 cortex; 6 animals; *n* = 10 neurons), the induction train evoked an LTD in control mice. Thirty minutes after the induction train, whisker responses reached 73.7 ± 28.68% of basal values in the presence of wortmannin, while in control conditions, it was 186.5 ± 30.21% (*n* = 10 neurons; Figure 4A). In the presence of rapamycin, whisker responses reached 62.7 ± 10.37% at 30 min after the induction train of basal values in comparison with control responses (Figure 4B). Differences respect to control values were statistically significant in presence of wortmaninn [Two-Way RM ANOVA, group factor: *F* (1, 224) = 14.50 *p* = 0.0002 ***] or rapamycin [Two-Way RM ANOVA, group factor: *F* (1, 259) = 19.56 *p* < 0.0001 ***]. Thus, the data suggested that the synaptic behavior observed in diabetic animals may be due to a reduction of the intracellular PI3K-AKT–mTOR pathway, since IGF-I signaling was also reduced in these animals.

### 3.5 Immunohistochemical studies

**Figure 5:**
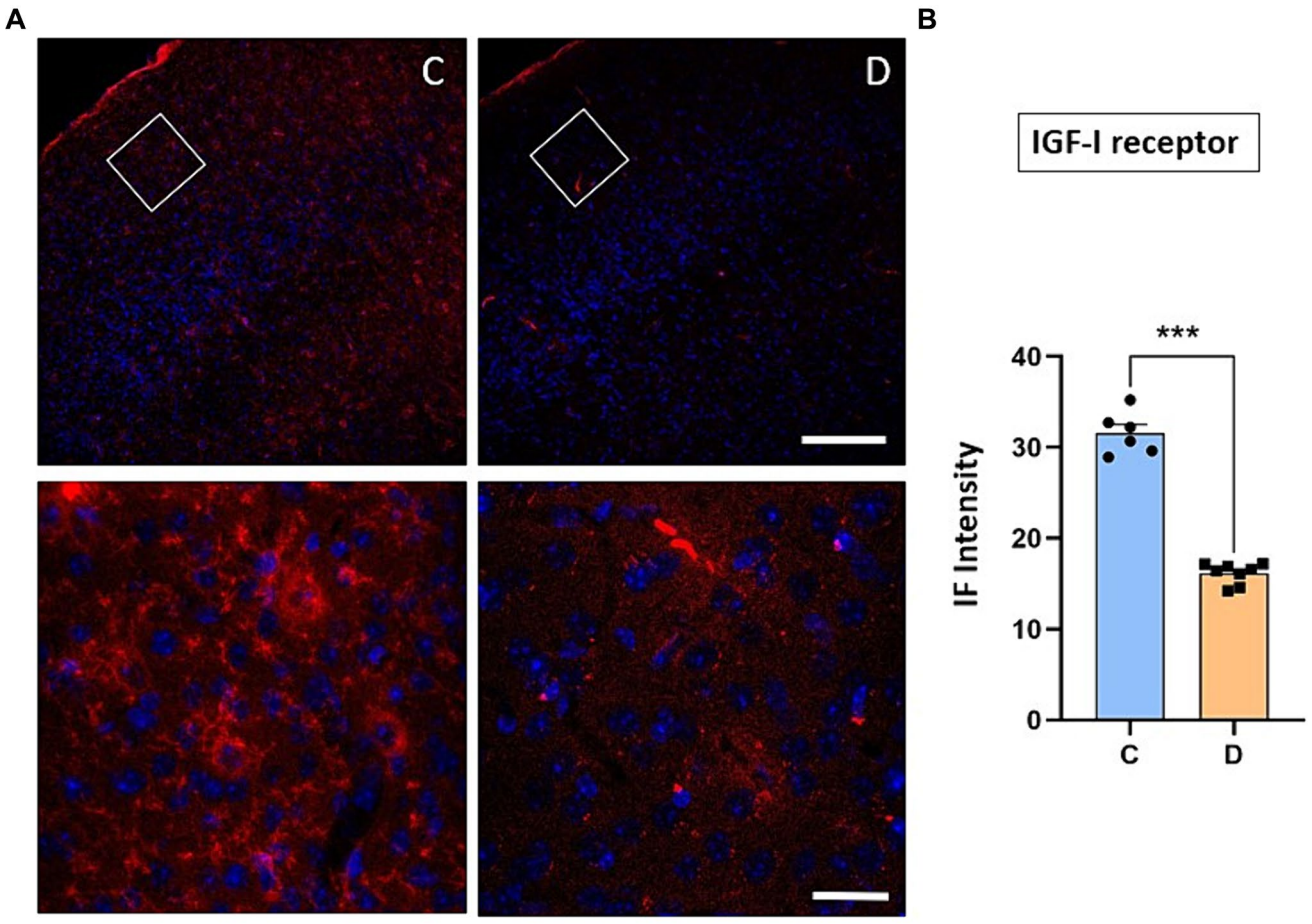
Immunohistochemical location of IGF-I receptors in the S1 cortex. **(A)** Representative photomicrographs of IGF-IR and Hoesch immunohistochemistry in the S1 cortex of control (left) and STZ-diabetic (right) animals at 20X (scale bar 150 μm; upper photomicrographs) or 63X (scale bar 20 μm; lower photomicrographs from the area represented by a white square). Supragranular layer (layer 2/3) showed higher expression of the IGF-IR in control animals. **(B)** Comparisons of densitometric measurements for IGF-IR in layers 2/3 of control and STZ-diabetic animals. Values of the optical density are represented as means ± SEM of the normalized fluorescence intensity (*p* < 0.0001). Scale bar 150 μm.

The immunohistochemical studies were focused on the 2/3 layers of S1 cortex in order to test if changes in synaptic transmission in the cortex could explain the changes in synaptic plasticity observed in STZ-diabetic mice. First, we observed that the immunodensity of the IGF-I receptor decreased by 51% in STZ-diabetic mice (4 animals) compared to control mice (4 animals), indicating that IGF-I signaling was reduced (Figure 5).

**Figure 6:**
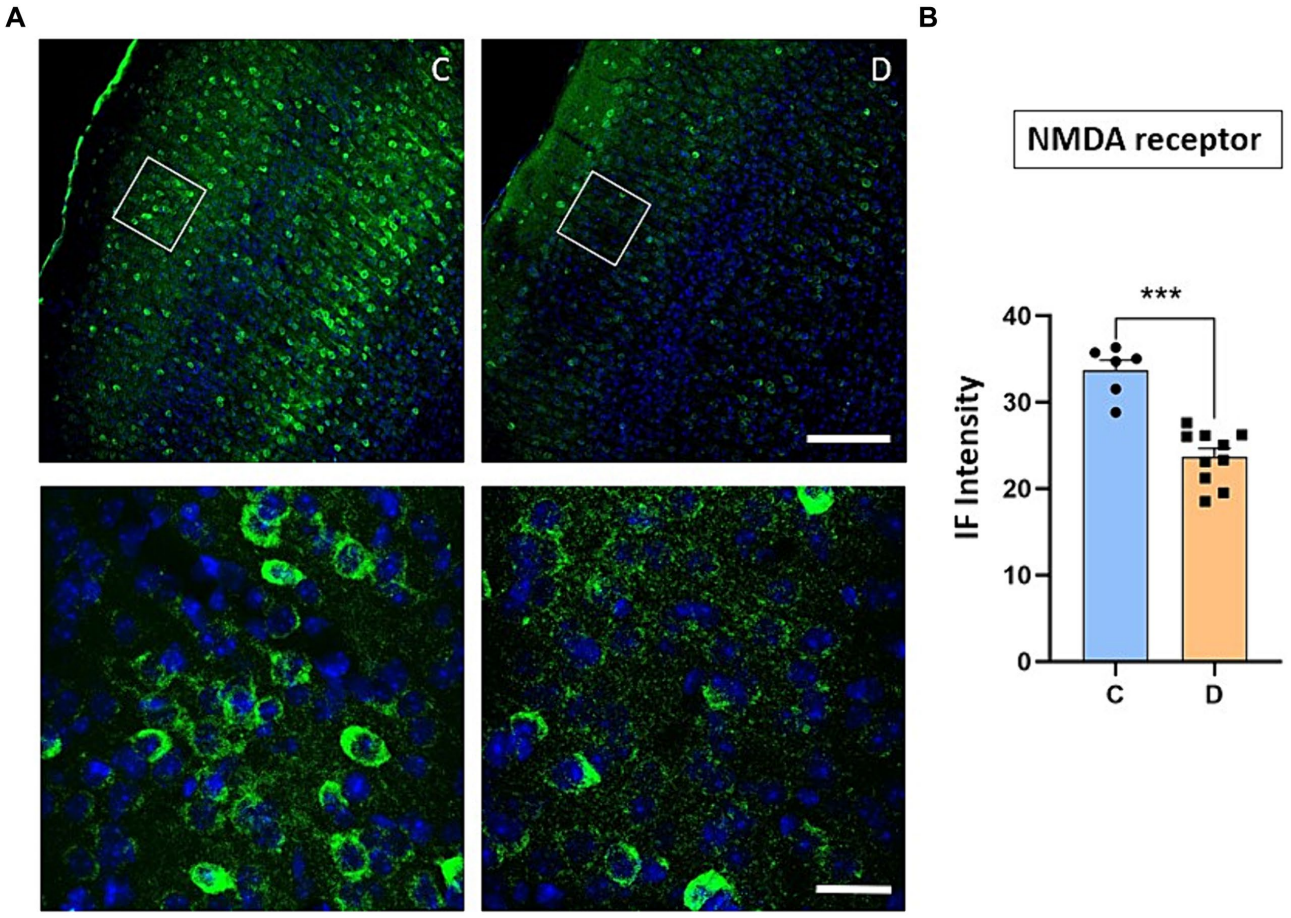
Confocal images show a noticeable decrease in NMDA receptor expression in STZ-diabetic animals. **(A)** Representative photomicrographs of NMDA receptors and Hoesch immunohistochemistry in the S1 cortex of control (left) and STZ-diabetic (right) animals at 20X (scale bar 150 μm; upper photomicrographs) or 63X (scale bar 20 μm; lower photomicrographs from the area represented by a white square). Supragranular layer (layer 2/3) showed lower expression of the NMDA receptors in STZ-diabetic animals respect to control animals. **(B)** Comparisons of densitometric measurements for NMDA receptors in layers 2/3 of control and STZ-diabetic animals. Values of the optical density are represented as means ± SEM of the normalized fluorescence intensity (*p* = 0.0002).

**Figure 7:**
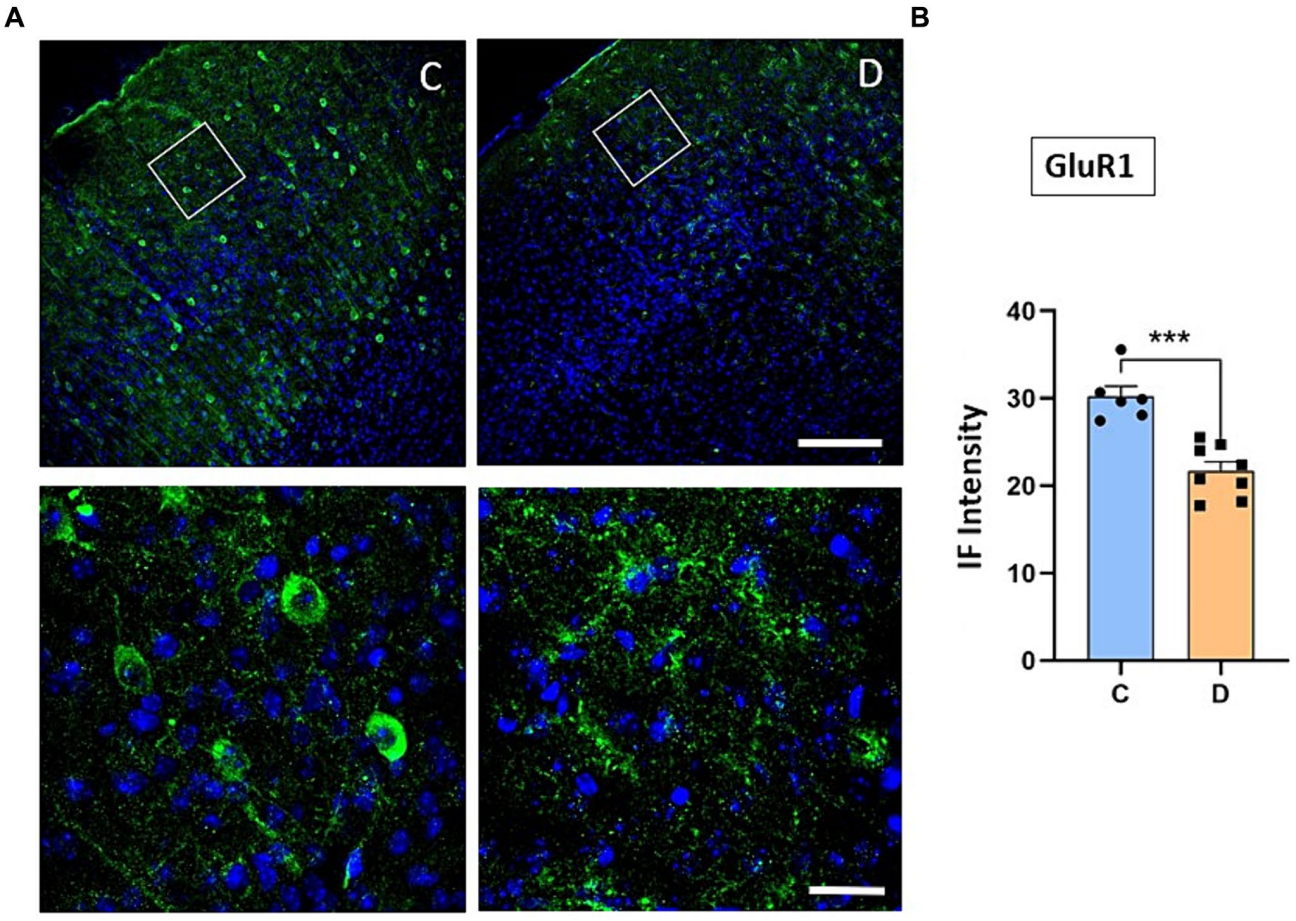
Confocal images show a decrease of GluR1 receptor expression in diabetic animals. **(A)** Representative confocal images of coronal sections of S1 cortex in control (left) and in STZ-diabetic mice (right) at 20X (scale bar 150 μm; upper photomicrographs) or 63X (scale bar 20 μm; lower photomicrographs from the area represented by a white square). **(B)** Comparisons of densitometric measurements show a significant decrease in GluR1 expression in STZ-diabetic mice compared to control mice (*p* < 0.0001).

We have indicated above that the IGF-I intracellular signaling pathway was affected in STZ-diabetic mice and thus, the PI3K-AKT–mTOR pathway activated by IGF-I could affect the incorporation of glutamatergic receptors to the cellular membrane and thus, explaining the change from LTP to LTD in STZ-diabetic mice. To test this hypothesis, we performed immunohistochemical studies of the NMDA and GluR1 receptors in the S1 cortex of control and in STZ-diabetic mice (4 mice in each animal group). Figure 6 shows that NMDAR immunodensity decreased up to 71% in STZ-diabetic mice compared to control mice. Similarly, immunodensity of GluR1 decreased up to 72% in STZ-diabetic mice (Figure 7).

### 3.6 STZ-diabetic mice showed impaired performance in a whisker discrimination task

**Figure 8:**
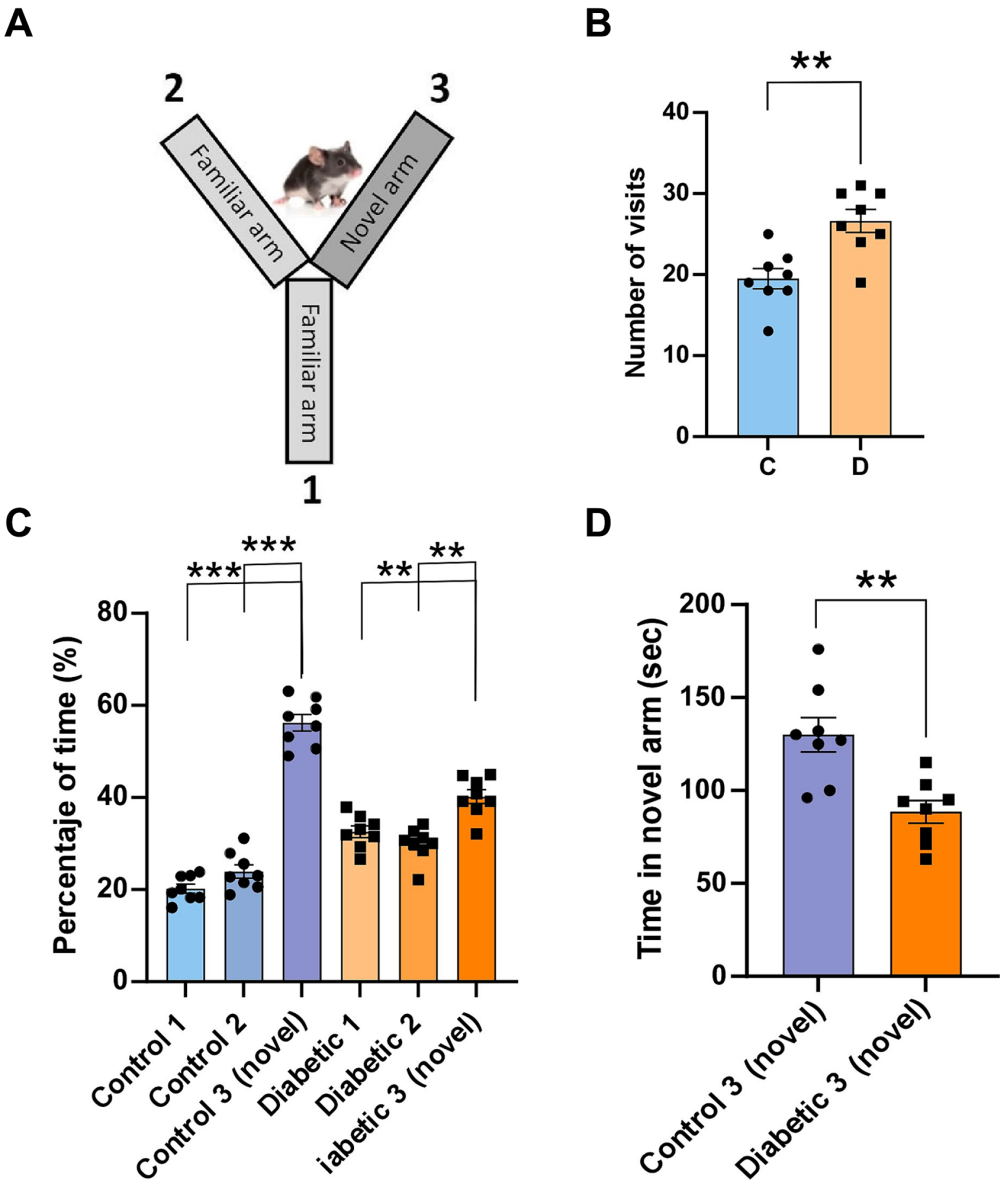
STZ-diabetic mice showed impaired the performance of a whisker discrimination task. **(A)** A scheme of the Y-maze (the walls of the maze arms were covered with two different grades of black sandpaper); the walls of two arms (1 and 2) were covered with 500-grit sandpaper (familiar texture), while the arm 3 was covered with 220-grit sandpaper (novel texture). **(B)** number of visits of all arms for control and STZ-diabetic mice. STZ-diabetic mice show large number of visits than control mice. **(C)** Percentage of time spent in each arm (1–3). Control mice visit more frequently the novel arm than STZ-diabetic mice. **(D)** STZ-diabetic mice spent less time examining the arm with the new texture compared with the control mice.

We assessed the ability of the mice to discriminate between different textures in a Y-maze by comparing the behavior of the control group to that of the STZ-diabetic mouse group (Figure 8A). Mice discriminated between different textures in the arms of a Y-maze (the walls of the maze arms were covered with two different grades of black sandpaper), as well as the spatial working memory since mice with good short-term memory remember the arms they have previously visited. STZ-diabetic mice showed larger number of visits of all arms than control mice (19.5 ± 1.2 s vs26.6 ± 1.4; *p* = 0.002; *n* = 8; Figure 8B). However, if we studied the percentage of time that mice spent in each arm, we observed that control mice tend to visit more time in the novel C arm that the others (20.2 ± 0.9%; 23.9 ± 1.4% and 56.3 ± 1.8%; arms A-C, respectively; *p* < 0.001, arm C respect to A and B arms; *n* = 8; Figure 8C). STZ-diabetic mice also discriminated the novel C arm, but differences were lower (32.6 ± 1.3%; 29.9 ± 1.3% and 40.2 ± 1.5%; arms A-C, respectively; *p* = 0.0086 and *p* = 0.0026 A and B arms respect C arm; *n* = 8). Thus, STZ-diabetic mice spent less time examining the arm with the new texture compared with the control mice (130 ± 9.2 s vs. 88.5 ± 6.1 s; *p* = 0.002; *n* = 8; Figure 8D), indicating that STZ-diabetic mice animals have deteriorated texture discrimination.

## 4 Discussion

The present results demonstrate significant changes in synaptic plasticity in the S1 cortex of STZ-diabetic mice. Under control conditions, repetitive stimulation of whiskers (8 Hz induction train) induced LTP in layer 2/3 neurons. Conversely, in STZ-diabetic mice, the same induction train led to LTD, which was dependent on NMDA and metabotropic glutamatergic receptors. This change in synaptic plasticity may be due to the reduction in IGF-I brain levels that occurs in diabetic animal models and patients (Ishii et al., 1994; Guo et al., 1999; Lupien et al., 2003). Response facilitation was enhanced in STZ-diabetic mice when IGF-I was administered 15 min before the induction train. This hypothesis was further supported by immunochemical techniques, which revealed a reduction of IGF-I receptors in layer 2/3 of the S1 cortex in STZ-diabetic animals. The observed impairments in synaptic plasticity in STZ-diabetic animals were accompanied by a decline in performance on a whisker discrimination task. Moreover, in control animals, the induction train produced LTD instead of LTP when a non-selective phosphoinositide kinase inhibitor, wortmannin, or rapamycin, an mTOR inhibitor, was applied to the S1 cortex. These findings suggest that the synaptic plasticity deficits in STZ-diabetic mice stem from reduced activation of IGF-I receptors in diabetes, leading to decreased activity in the PI3K-mTOR signaling pathway and consequent reduction in the incorporation of glutamatergic receptors into the membrane. This decrease in PI3K-mTOR signaling pathway activity could explain the shift from LTP in control mice to LTD in STZ-diabetic mice. Consistent with this hypothesis, immunochemical studies revealed reductions in IGF-I, GluR1, and NMDA receptors.

This study has been limited to the study of male mice because female mice show greater resistance to the toxin compared to male mice. Consequently, most studies using the STZ-induced diabetic model have been conducted with male animals (Chandramouli et al., 2018; Furman, 2021). Another limitation concerns the type of anesthesia used in the experiments. Isoflurane has been observed to increase inhibition mediated by GABAergic neurons in several studies, which may affect synaptic plasticity (Kotani and Akaike, 2013; Long Ii et al., 2016). In addition, it is known that isoflurane impairs insulin secretion and glucose utilization (Tanaka et al., 2009). However, we do not believe that our findings are due to the anesthesia, considering that both experimental groups were exposed to the same anesthesia conditions; differences in synaptic plasticity must be due to the presence of diabetes since differences were also observed in the immunohistochemical studies that were not affected by the anesthesia. However, it is necessary to consider the influence of anesthetics in future research.

Previous findings suggest that both type 1 and type 2 DM are associated with cognitive impairment (Kamal et al., 2000; Artola, 2013; Saedi et al., 2016; Li et al., 2017). For example, a reduction of learning, memory, and mental flexibility, are common in patients with type 1 DM (Ryan, 1988; Biessels and Gispen, 2005; Shalimova et al., 2019). These cognitive deficits have also been observed in animal models of diabetes. For instance, young-adult STZ-diabetic rats begin to exhibit learning deficits in the Morris maze approximately 10 weeks after the induction of diabetes (Biessels et al., 1996; Kamal et al., 2000; Valastro et al., 2002).

The two major forms of long-lasting synaptic plasticity in the mammalian brain, known as LTP and LTD, are characterized by a persistent increase or decrease in synaptic strength, respectively. Both processes are believed to play crucial roles in information storage and, consequently, in learning and memory functions. Thus, it is reasonable to hypothesize that impairment of synaptic plasticity could contribute to cognitive deficits in diabetes. Indeed, we have demonstrated a significant alteration in synaptic plasticity in STZ-diabetic mice. In control rats (Barros-Zulaica et al., 2014) or mice (Garcia-Magro et al., 2022), repetitive stimulation of whiskers induce LTP in the S1 cortex, as also occurred in present results. However, we observed that the same stimulation protocol induced LTD in STZ-diabetic mice. It is widely accepted that the induction of LTP in the hippocampus and neocortex requires the activation of postsynaptic NMDA receptors. This activation allows the influx of a substantial amount of Ca2+ into the cell, which is necessary to trigger LTP (Artola and Singer, 1993; Bear and Malenka, 1994; Fino et al., 2008). In agreement with that, the LTP observed in our study in control mice was blocked by local application of the NMDA receptor antagonist AP5. Previous findings have indicated that LTD is triggered by synaptic activation of either NMDA or metabotropic glutamate receptors (Stanton et al., 2003; Zhang et al., 2006; Collingridge et al., 2010; Rodriguez-Moreno et al., 2013). Consistent with those studies, our findings in STZ diabetic mice revealed that NMDA and metabotropic glutamate receptors were involved in the generation of LTD because local application of AP5 or the non-selective metabotropic glutamate receptor antagonist MCPG reduced whisker-evoked LTD, although it was not possible to restore the LTP shown by control mice.

Alterations in synaptic plasticity have been observed in hippocampal slices from diabetic rats (Valastro et al., 2002). For example, diabetic rats exhibited reduced LTP after high-frequency stimulation, while low-frequency stimulation induced a larger LTD (Biessels et al., 1996; Chabot et al., 1997; Kamal et al., 2000, 2005). Both LTP and LTD in the hippocampus require activation of NMDA receptors, increasing postsynaptic intracellular [Ca2+] (Malenka and Nicoll, 1993; Malenka and Bear, 2004). The critical event which determines whether stimulation of NMDA receptors and subsequent Ca2+ influx leads to either LTD or LTP appears to be the magnitude and the pattern of the Ca2+ signal (Lisman, 1989; Artola et al., 1990; Mulkey and Malenka, 1992; Malenka and Nicoll, 1993). Thus, high intracellular [Ca2+] is thought to activate protein kinases necessary for LTP and low [Ca2+] activate protein phosphatases necessary for LTD (Mulkey and Malenka, 1992; Malenka and Nicoll, 1993; Nabavi et al., 2014). Therefore, diabetes might disturb this delicate balance of intracellular [Ca2+], explaining the transition from LTP to LTD that we observed here in STZ-diabetic mice. In agreement with this, our findings suggest that LTD was elicited by repetitive stimulation in STZ-diabetic mice due to reductions in NMDA and GluR1 receptors in S1 cortical cells. However, other authors have indicated that in the hippocampus, there is an up-regulation of NMDA and AMPA receptors in early stages of diabetes, together with a reduction in the induction of LTP in hippocampal slices. Nevertheless, a significant alteration in glutamate receptors in the parieto-temporal cortex was not found (Valastro et al., 2002). These differences may arise from the varying impact of diabetes on the hippocampus and neocortex, given their distinct synaptic inputs. Additionally, the discrepancies could be attributed to differences in the studied populations. The previous study examined changes in glutamate receptors in spontaneous diabetic mice aged 12–30 old weeks, whereas our experiments were conducted in younger animals aged 12 old weeks, in which diabetes was induced by STZ.

Several authors have suggested that these changes in plasticity may reflect alterations in intracellular signaling pathways. For example, Jolivalt and colleagues indicated that mice with systemic insulin deficiency displayed evidence of reduced insulin-signaling pathway activity and reduced phosphorylation of the insulin-receptor; treatment with insulin partially restored these changes (Jolivalt et al., 2008). The present results demonstrate that repetitive whisker stimulation induces LTD in control mice when the PI3K signaling pathway was blocked with local application of wortmannin (a non-selective phosphoinositol kinase inhibitor) or with rapamycin, an mTOR inhibitor. Previous findings support a role for PI3-Kinase/Akt/ mTOR-dependent signaling pathway in learning processes (Kelly and Lynch, 2000; Bailey et al., 2012). The role of PI3-kinase in the maintenance or expression of LTP is also supported by the absence of LTP in the hippocampus of animals pretreated with wortmannin (Kelly and Lynch, 2000), or in the prefrontal cortex (Sui et al., 2008) or in the basolateral amygdala (Lin et al., 2001). Moreover, Western blot analysis has revealed that pAkt/Akt, pmTOR/mTOR and pPI3K/PI3K were decreased in the cerebral cortex of STZ-induced type 2 DM (Bathina and Das, 2018). Besides, activating mTOR/NF-κB signaling pathway plays a critical role in the pathogenesis of diabetic encephalopathy, including neuroinflammation, synaptic protein loss, and synaptic ultrastructure impairment (Xu et al., 2021).

In addition, PI3-kinase activity is involved in membrane trafficking in the hippocampus (Sanna et al., 2002). Hence, PI3-kinase may be implicated in signaling mechanisms that regulate AMPA receptor density at postsynaptic sites. Current understanding suggests that the mechanisms underlying synaptic plasticity involve changes in the density of AMPA receptors on postsynaptic membranes (Malenka and Nicoll, 1993; Lüscher et al., 2000; Malinow and Malenka, 2002; Diaz-Alonso et al., 2020; Diaz-Alonso and Nicoll, 2021). In the hippocampus, LTP is characterized by a response increase of AMPA receptors (Malenka and Nicoll, 1993, Malinow and Malenka, 2002). AMPA receptors with varying subunit compositions seem to underlie basal AMPA responses as well as the enhanced AMPA-mediated transmission observed during LTP (Chung et al., 2000; Lüscher et al., 2000; Scannevin and Huganir, 2000; Malinow and Malenka, 2002; Fernandez de Sevilla et al., 2008). Previous results have indicated an important role for the GluR1 subunit in hippocampal LTP since GluR1−/− mice do not show LTP (Zamanillo et al., 1999), and GluR1 receptors are incorporated into synapses during LTP (Hayashi et al., 2000). Here, we observed that the immunostaining of GluR1 AMPA receptors was reduced in the S1 cortex of STZ-diabetic mice, suggesting that this reduction in GluR1 could contribute to the change from LTP in control animals to LTD in STZ-diabetic animals. Previously, we have shown a reduction of the sciatic nerve evoked potential amplitude in S1 cortex in diabetic rats (Piriz et al., 2009). This decrease was accompanied by reduction in GluR2/3 AMPA receptor subunits. Thus, the change in glutamatergic AMPA and NMDA receptors on the postsynaptic membrane may be the cause of altered synaptic plasticity in diabetes.

The findings observed in STZ-diabetic mice here may be attributed to a reduction in IGF-I signaling. The whisker-evoked LTD was replaced by response facilitation after local injection of IGF-I, although LTP was not completely restored as in control animals. These data suggest that the decrease in the number of IGF-I receptors in the cortex may be responsible for the alteration in whisker-evoked synaptic plasticity. In agreement with these findings, the same induction train evoked an LTD in a mouse model of serum IGF-I deficiency which show a reduced brain IGF-I input (LID mice; see Figure 1D) (Trueba-Saiz et al., 2013). A reduction in IGF-I and insulin levels has been proposed as a potential cause of neurological disorders in diabetes, as administration of IGF-I can prevent cognitive decline (Ishii and Lupien, 1995; Guo et al., 1999; Ishii and Lupien, 2003; Zegarra-Valdivia et al., 2019). All major components of the PIK3 pathway (IRS1/2, PI3K, AKT) and constituents of the MAPK cascade are involved in both insulin and IGF-I signaling (Endo et al., 2006). In agreement with this, we observed a decrease in IGF-I receptor immunostaining in layer 2/3 neurons of the S1 cortex in STZ-diabetic mice compared to control mice. Synaptic plasticity. Above results suggest that the reduction of activation of IGF-I receptors, and probably insulin receptors, in T1DM produces changes in intracellular signaling pathways that cause a decrease in Ca2+ entry due to synaptic inputs and, therefore, changes in synaptic plasticity. Consequently, facilitation of LTD and inhibition of LTP may contribute to learning and memory impairments associated with DM (Artola et al., 2005).

In conclusion, we have demonstrated that T1DM mice induced by STZ injection exhibit impaired synaptic plasticity in the S1 cortex. The fact that IGF-I partially restore cognitive impairment observed in neurodegenerative diseases such as diabetes (present results) or Alzheimer’s disease (Zegarra-Valdivia et al., 2019), and in aging (Garcia-Magro et al., 2022) suggest that the reduction of IGF-I signaling may be involved in these processes. Further studies are needed to investigate the cellular mechanisms implicated in IGF-I signaling and the influence of neurodegenerative diseases on these processes.

## Data availability statement

The raw data supporting the conclusions of this article will be made available by the authors, without undue reservation.

## Ethics statement

The animal study was approved by the Ethical Committee of the Autonomous University of Madrid and Government of the Community of Madrid (PROEX: 181.6/21). The study was conducted in accordance with the local legislation and institutional requirements.

## Author contributions

NG-M: Writing – review & editing, Writing – original draft, Methodology, Investigation, Formal analysis. AM-L: Writing – review & editing, Writing – original draft, Methodology, Investigation, Formal analysis. NB-Z: Writing – review & editing, Writing – original draft, Software, Formal analysis. ÁN: Writing – review & editing, Writing – original draft, Supervision, Project administration, Funding acquisition, Conceptualization.
